# Supplementary material for: Estimation and implications of the genetic architecture of fasting and non-fasting blood glucose
Source: Nat Commun. 2023 Jan 27;14:451. doi: 10.1038/s41467-023-36013-1 (PMC9883484; doi:10.1038/s41467-023-36013-1)
Supplement: Supplementary file 1 — Supplementary Information [file 41467_2023_36013_MOESM1_ESM.pdf]

Supplementary Material

Estimation and implications of the genetic architecture of fasting and non-fasting blood glucose

Qiao et al.

SUPPLEMENTARY METHODS **2**

Sample Selection Criteria **2**

SUPPLEMENTARY FIGURES **3**

ACKNOWLEDGEMENTS **17**

GROUP AUTHORS **17**

SUPPLEMENTARY REFERENCES **18**

SUPPLEMENTARY METHODS

Sample Selection Criteria

UKB samples were excluded if they were diagnosed as any subtypes of diabetes, on diabetes medications, had abnormal glucose (≥ 3mmol/L or ≤ 11.1 mmol/L) or glycated haemoglobin (HbA1c ≥ 48 mmol/mol) levels in any visits to the assessment centre. We further restricted our analyses to European samples. Inference of sample ancestry were described in Yengo et al[1].

To be specific, individuals with diabetic conditions were identified through the following procedures,

1. We used first occurrences of health-related outcomes (Category 1712) as a first pass to exclude individuals with diabetic conditions. This category encodes data by mapping the information from primary care data (Category 3000), hospital inpatient data (Category 2000), death register records (Field 40001-40002) and self-reported medical condition codes (Field 20002) to International Classification of Diseases-10 (ICD-10) codes. UKB samples with all diabetes mellitus diseases, including E10 (insulin-dependent diabetes mellitus, T1D), E11 (non-insulin-dependent diabetes mellitus, T2D), E12 (malnutrition-related diabetes mellitus), E13 (other specified diabetes mellitus), E14 (unspecified diabetes mellitus) and O24 (diabetes mellitus in pregnancy), were excluded from downstream analyses (*N*= 40,401 were excluded).

Since Category 1712 has not been externally validated, we further investigated the following data-fields to remove diabetic samples.

2. We examined the summary diagnosis hospital inpatient data encoded according to ICD-9 (Data-Field 41271) and ICD-10 (Data-Field 41270) and excluded samples diagnosed with diabetic diseases (coding listed in Supplementary Data 1).
3. We also examined self-reported non-cancer illness (Data-Field 20002) and excluded samples with self-reported diabetic diseases, including diabetes, gestational diabetes, T1D and T2D (1220-1223, Data-Coding 6).
4. We then checked the death register (Data-Field 40023), in which the cause of death was encoded following ICD-10. We excluded individuals with recorded cause of death due to diabetic diseases in Supplementary Data 1.
5. We further excluded individuals who reported to use diabetes-relevant medications based on Data-Field 6153 (Data-Coding 100626, "Insulin"), Data-Field 6177 (Data-Coding 100625, "Insulin") and Data-Field 20003 (Data-Coding 4, treatment/medication for diabetes in this data field were extracted and listed in Supplementary Data 1).
6. Lastly, we excluded individuals with potential diabetic conditions by examining the following data-fields, including diabetes diagnosed by doctors (Data-Field 2443), age diabetes diagnosed (Data-Field 2976), started insulin within one year diagnosis of diabetes (Data-Field 2986), gestational diabetes only (Data-Field 4041), and age when diabetes-related eye disease diagnosed (Data-Field 5901).

SUPPLEMENTARY FIGURES

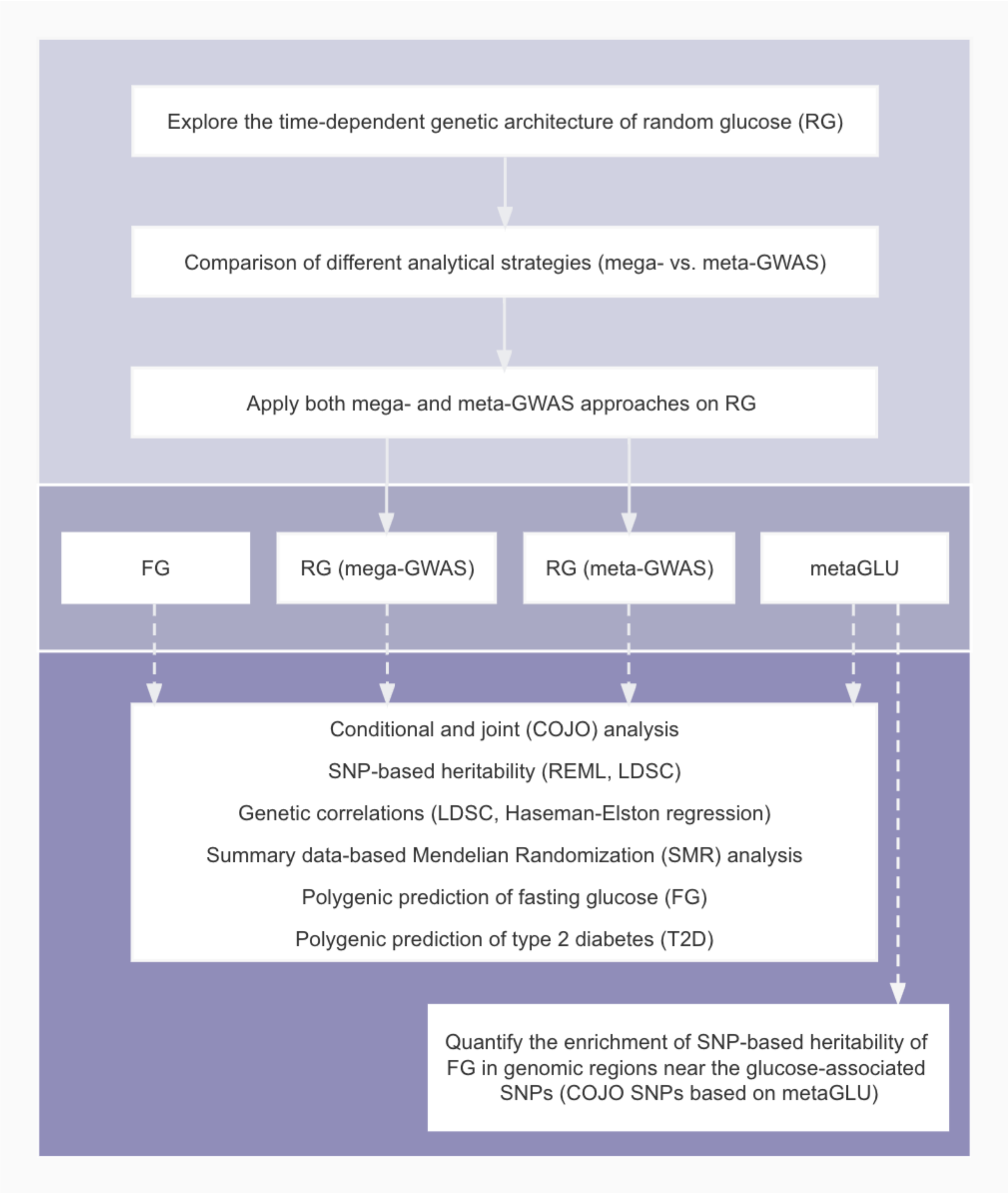

Supplementary Figure 1. Overview of the study design. The four GWAS summary statistics used in this study: FG is the summary statistics of fasting glucose from Lagou et al. (2021)[2]; mega-GWAS of RG and meta-GWAS of RG are the summary statistics of random glucose modelled by two different statistical approaches using UKB samples; metaGLU is the summary statistics by meta-analyzing the fasting glucose from Lagou et al. (2021) and random glucose from the current study (meta-GWAS of RG).

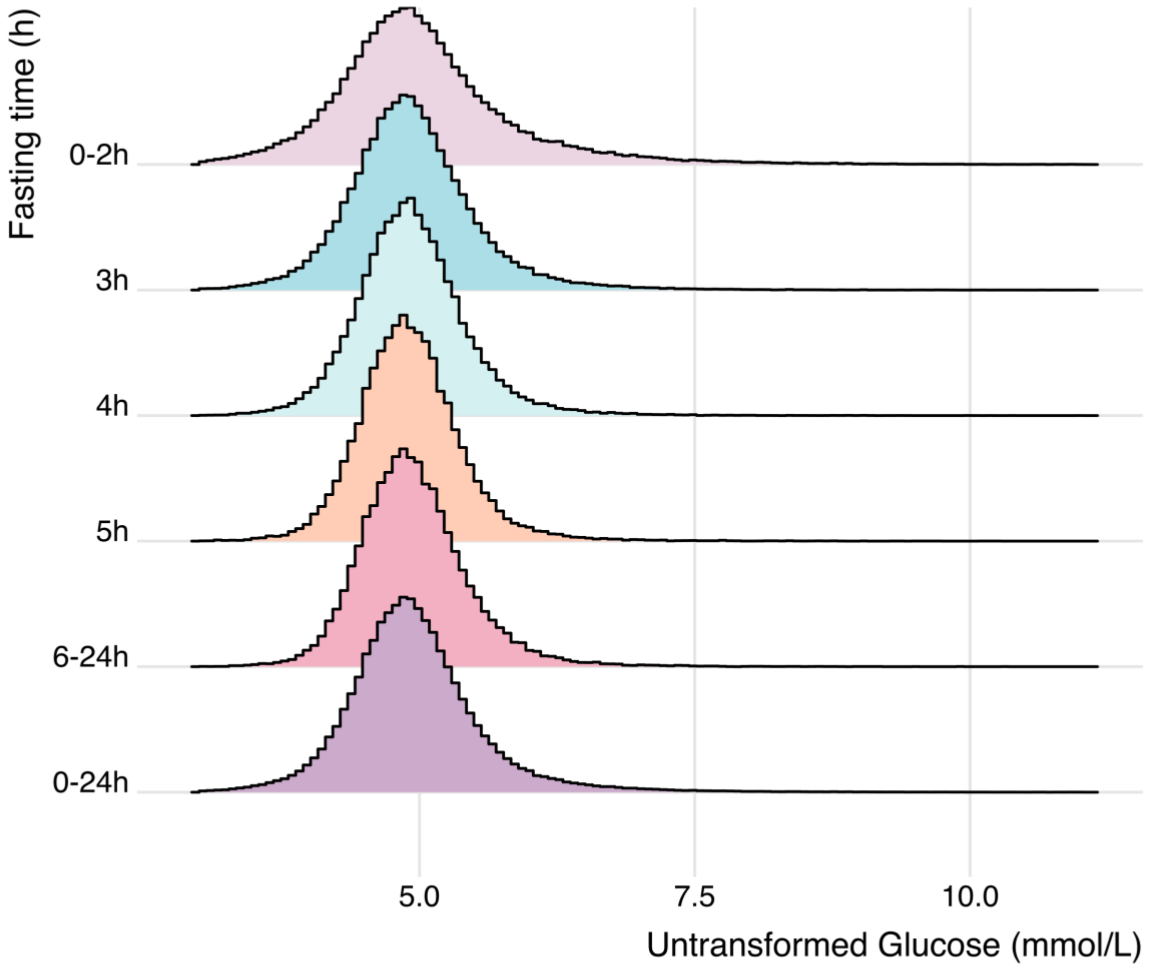

Supplementary Figure 2. Distribution of untransformed RG in each subgroup in the UK Biobank sample included in this study. Similar as previous MAGIC's studies, we used untransformed RG for our main analyses, which facilitated the comparison with previous results and made the results easier for interpretation (i.e., effect size in original units, mmol/L). RG glucose values > 11 mmol/L were excluded as part of quality control.

a

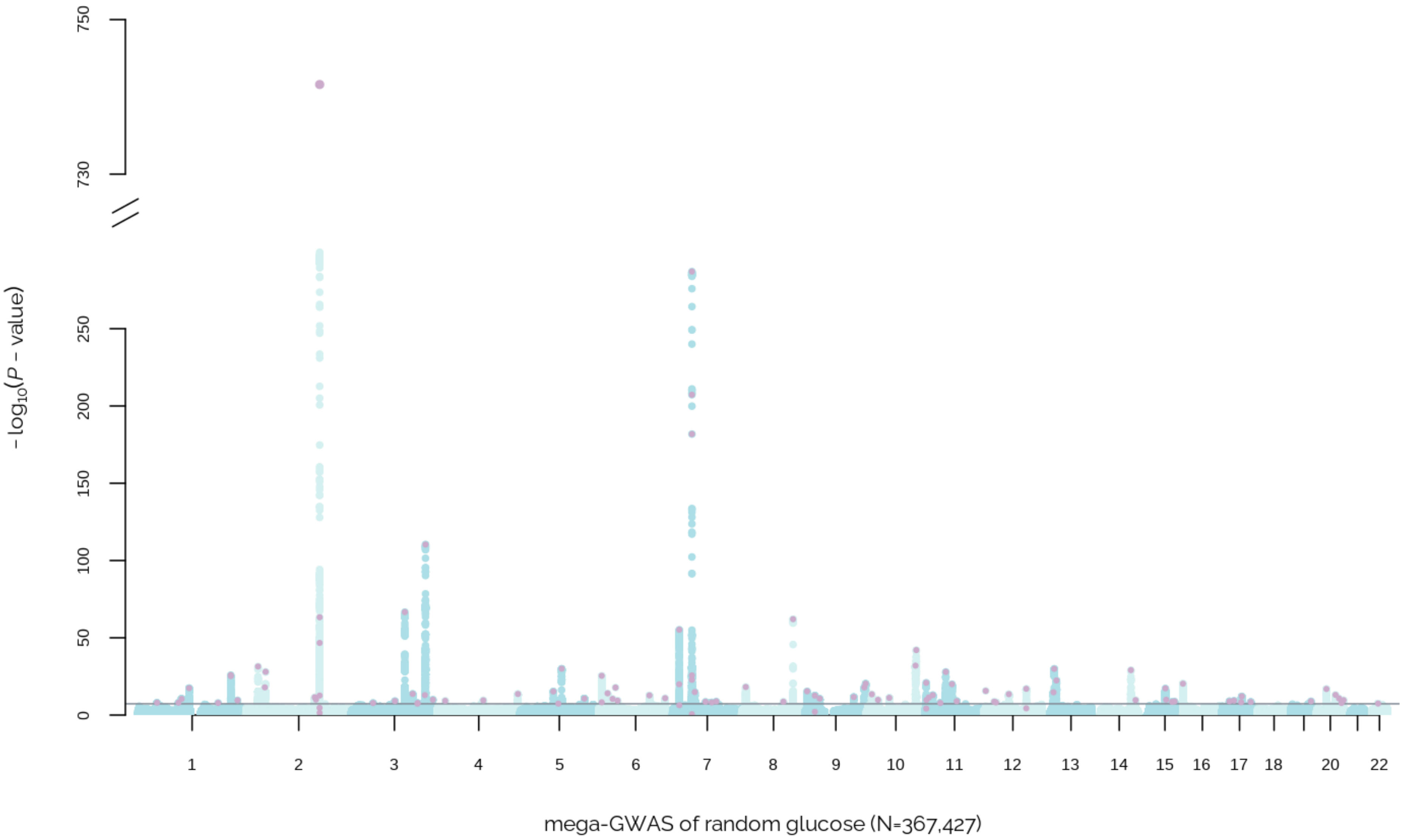
750
730
250
200
150
100
50
0
- log10(P - value)
1
2
3
4
5
6
7
8
9
10
11
12
13
14
15
16
17
18
20
22
mega-GWAS of random glucose (N=367,427)

b

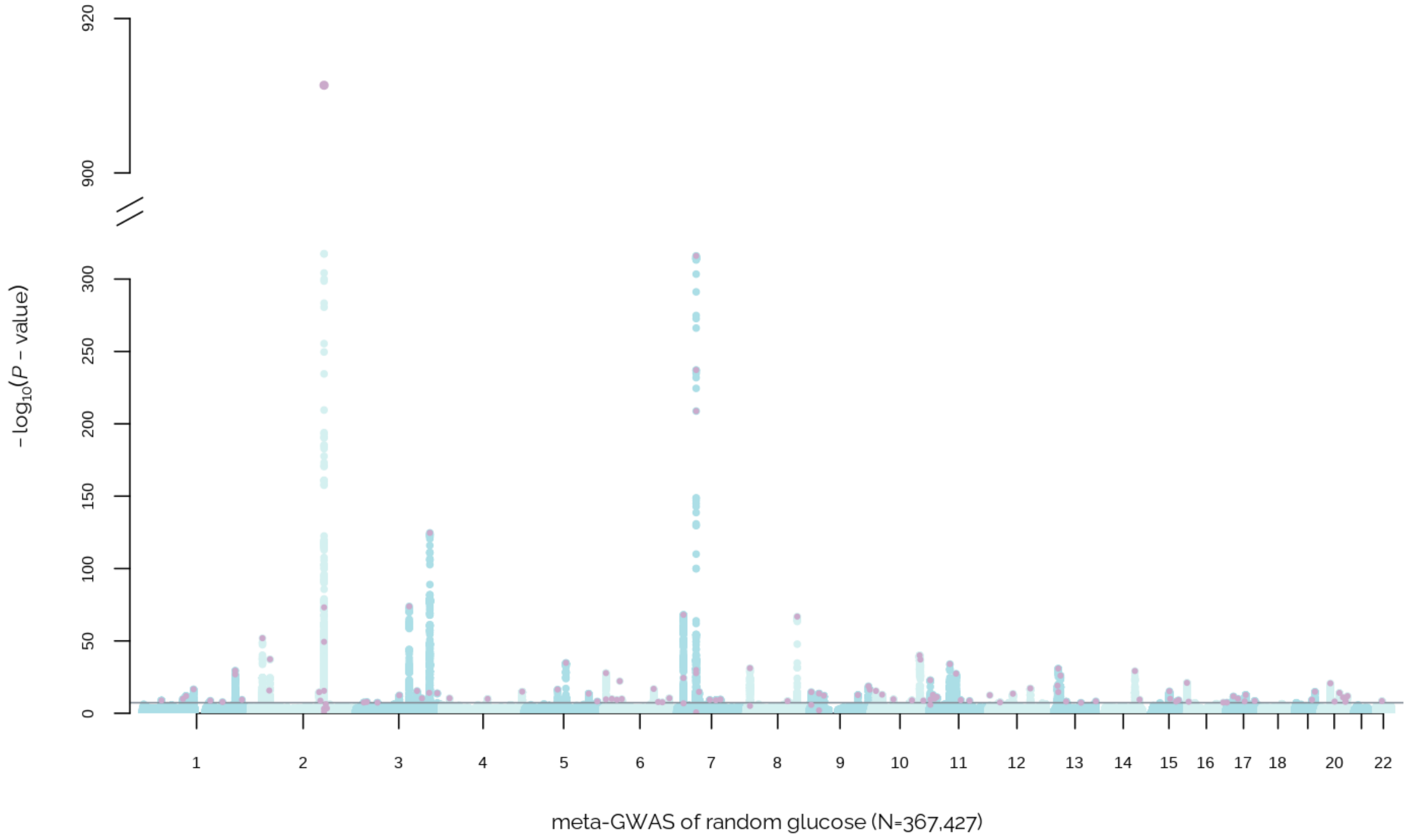
920
900
300
250
200
150
100
50
0
-log10(P - value)
1
2
3
4
5
6
7
8
9
10
11
12
13
14
15
16
17
18
20
22
meta-GWAS of random glucose (N=367,427)

c

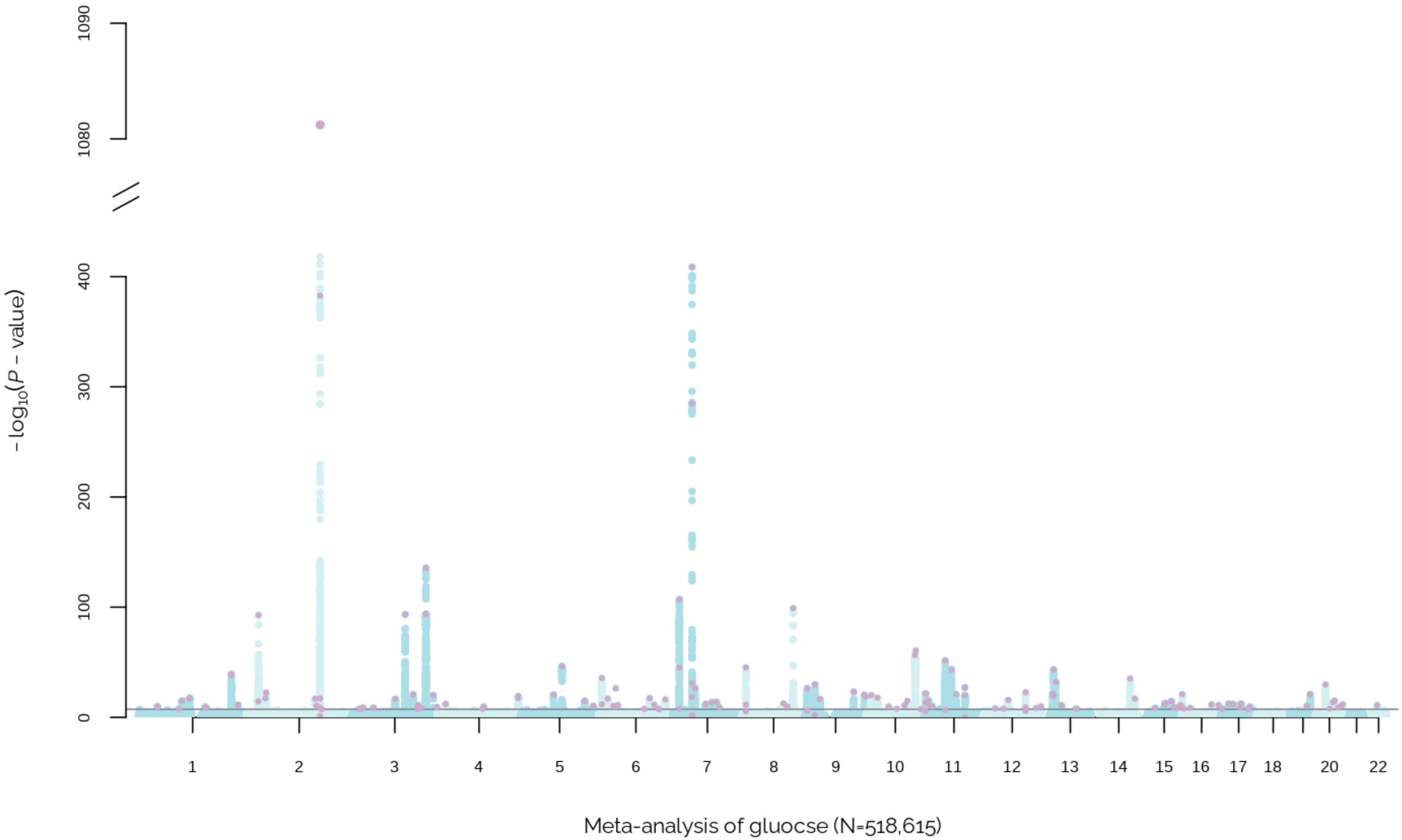

Supplementary Figure 3. Manhattan plots for the autosomal GWAS of mega-GWAS of random glucose (**a**), meta-GWAS of random glucose (**b**), and meta-analysis of glucose (**c**), depicting the -$\log_{10}$ *P*-values (two-tailed test) from BOLT-LMM mixed model association test (**a**) or inverse-variance weighted meta-analysis (**b** and **c**) with genome-wide SNPs. Purple dots represent independent common variants identified as genome-wide significant

(P_{COJO} < 5×10^{-8}) with GCTA-COJO[3] analysis applied to the GWAS summary statistics. The grey horizontal lines represent the genome-wide significant threshold of $P < 5\times10^{-8}$ (two-sided P-value). All analyses were restricted to autosomes (chromosome 1-22 on x axis) and common SNPs (MAF ≥ 0.01).

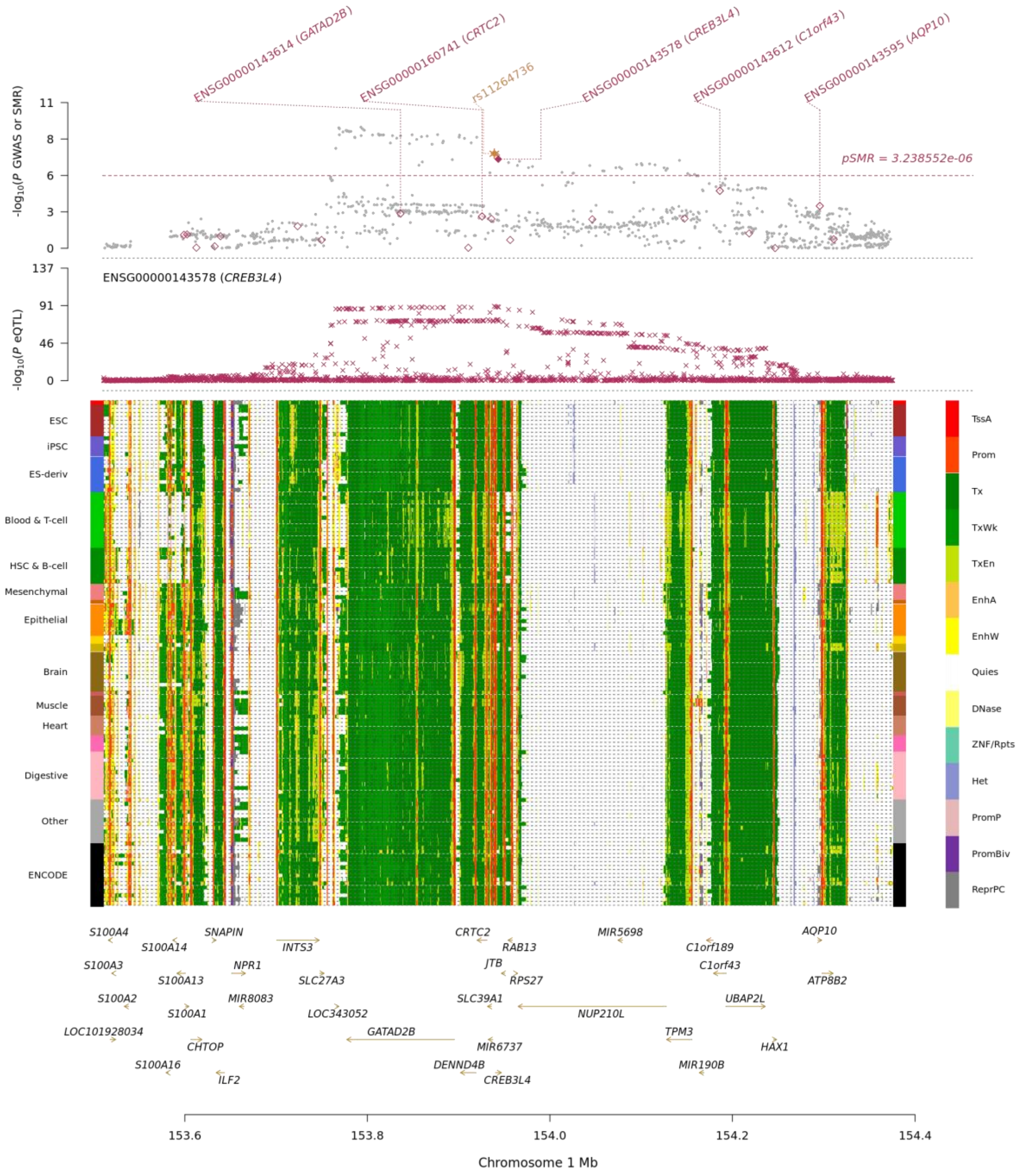

Supplementary Figure 4. SMR analyses and HEIDI tests that combined data from GWAS and eQTL studies. The first track displays -log$_{10}$(P-values) of the SNP associations (grey dots) from the meta-GWAS of random glucose (RG). The red rhombus represents -log$_{10}$(P-value) for probes from the SMR test and a solid rhombus indicate probes that passed the HEIDI test (i.e., *CREB3L4* passed the SMR and HEIDI test) with Bonferroni-corrected $P_{SMR} < 3.24\times10^{-6}$ (i.e., 0.05/m, with m = 15,439, being the total number probes tested in the SMR analysis) and $P_{HEIDI} > 0.01$. The yellow asterisk marks the top cis-eQTL (*rs11264736*). The second track displays -log$_{10}$(P-values) of the SNP associations from the eQTLGen study[4] for the ENSG00000143578 probe tagging *CREB3L4*. The third track displays the 25 chromatin state annotations (right bar) of 127 samples from the Roadmap Epigenomics Mapping Consortium (REMC)[5] for different tissues and cell types (left bar). The bottom track displays the genes underlying

the genomic region. Amongst the eQTLs tested, *rs11264736* is an eQTL for *CREB3L4* in blood and generates significant SMR associations to RG.

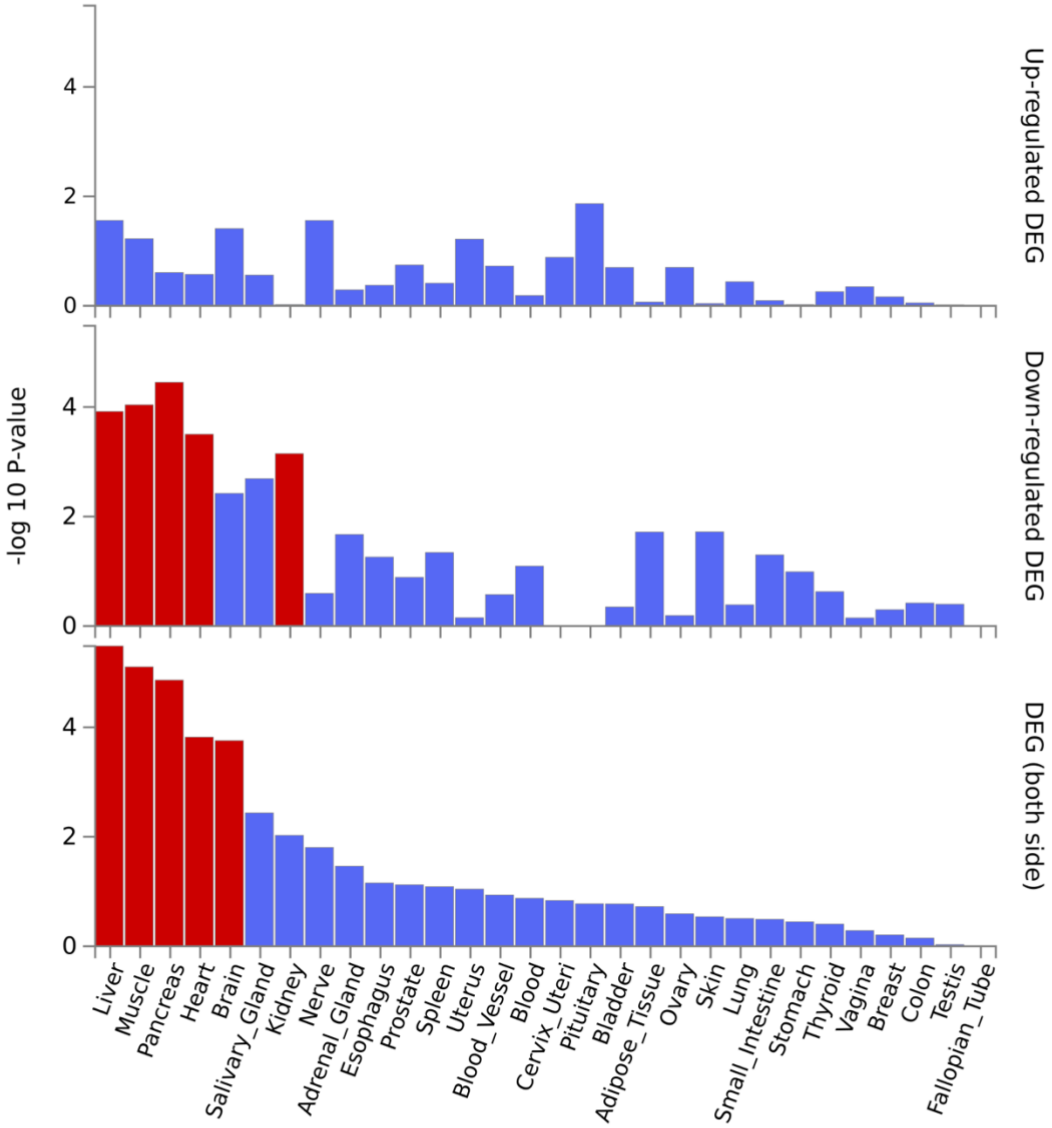

Supplementary Figure 5. FUMA GENE2FUNC differentially expressed genes (DEGs) output drawing upon GTEx v8 data for 30 general tissue types. The input genes were the SMR prioritized genes based on eQTL summary data from eQTLGen, InsPIRE and GTEx consortium for meta-analysis of glucose (metaGLU, number of genes=185). DEG sets were pre-calculated for each of 30 tissue type by conducting two-sided t-tests for each gene and tissue type against all other tissue types. The input genes were tested against each of the pre-calculated DEG sets defined for each of the selected tissues using hypergeometric tests. Red bars denote significantly enriched DEG sets with FDR corrected *P*-value < 0.05 (Benjamini–Hochberg method).

a

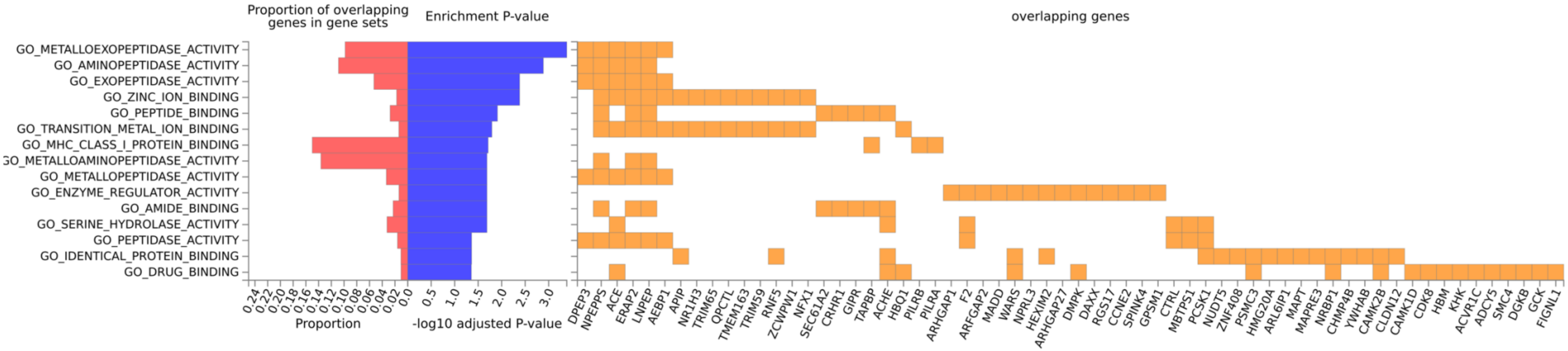

Proportion of overlapping genes in gene sets
Enrichment P-value
overlapping genes
GO_METALLOEXOPEPTIDASE_ACTIVITY
GO_AMINOPEPTIDASE_ACTIVITY
GO_EXOPEPTIDASE_ACTIVITY
GO_ZINC_ION_BINDING
GO_PEPTIDE_BINDING
GO_TRANSITION_METAL_ION_BINDING
GO_MHC_CLASS_I_PROTEIN_BINDING
GO_METALLOAMINOPEPTIDASE_ACTIVITY
GO_METALLOPEPTIDASE_ACTIVITY
GO_ENZYME_REGULATOR_ACTIVITY
GO_AMIDE_BINDING
GO_SERINE_HYDROLASE_ACTIVITY
GO_PEPTIDASE_ACTIVITY
GO_IDENTICAL_PROTEIN_BINDING
GO_DRUG_BINDING
Proportion
-log10 adjusted P-value
DPEP3 NPEPPS ACE ERAP2 LNPEP AEBP1 APIP NR1H3 TRIM65 QPCTL TMEM163 TRIM59 RNF5 ZCWPW1 NFX1 SEC61A2 CRHR1 GIPR TAPBP ACHE HBQ1 PILRB PILRA ARHGAP1 F2 ARFGAP2 MADD WARS NPRL3 HEXIM2 ARHGAP27 DMPK DAXX RGS17 CCNE2 SPINK4 GPSM1 CTRL MBTPS1 PCSK1 NUDT5 ZNF408 PSMC3 HMG20A ARL6IP1 MAPT MAPRE3 NRBP1 CHMP4B YWHAB CAMK2B CLDN12 CAMK1D CDK8 HBM KHK ACVR1C ADCY5 SMC4 DGKB GCK FIGNL1

b

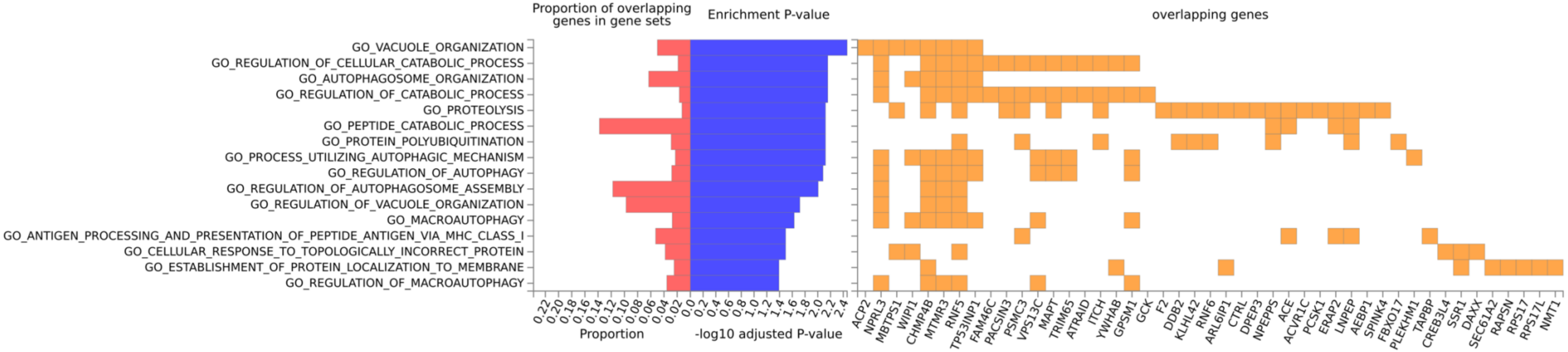

Proportion of overlapping genes in gene sets
Enrichment P-value
overlapping genes
GO_VACUOLE_ORGANIZATION
GO_REGULATION_OF_CELLULAR_CATABOLIC_PROCESS
GO_AUTOPHAGOSOME_ORGANIZATION
GO_REGULATION_OF_CATABOLIC_PROCESS
GO_PROTEOLYSIS
GO_PEPTIDE_CATABOLIC_PROCESS
GO_PROTEIN_POLYUBIQUITINATION
GO_PROCESS_UTILIZING_AUTOPHAGIC_MECHANISM
GO_REGULATION_OF_AUTOPHAGY
GO_REGULATION_OF_AUTOPHAGOSOME_ASSEMBLY
GO_REGULATION_OF_VACUOLE_ORGANIZATION
GO_MACROAUTOPHAGY
GO_ANTIGEN_PROCESSING_AND_PRESENTATION_OF_PEPTIDE_ANTIGEN_VIA_MHC_CLASS_I
GO_CELLULAR_RESPONSE_TO_TOPOLOGICALLY_INCORRECT_PROTEIN
GO_ESTABLISHMENT_OF_PROTEIN_LOCALIZATION_TO_MEMBRANE
GO_REGULATION_OF_MACROAUTOPHAGY
0.22 0.20 0.18 0.16 0.14 0.12 0.10 0.08 0.06 0.04 0.02 0.0
Proportion
0.2 0.4 0.6 0.8 1.0 1.2 1.4 1.6 1.8 2.0 2.2 2.4
-log10 adjusted P-value
ACP2 NPRL3 MBTPS1 WIPI1 CHMP4B MTMR3 RNF5 TP53INP1 FAM46C PACSIN3 PSMC3 VPS13C MAPT TRIM65 ATRAID ITCH YWHAB GPSM1 GCK F2 DDB2 KLHL42 RNF6 ARL6IP1 CTRL DPEP3 NPEPPS ACE ACVR1C PCSK1 ERAP2 LNPEP AEBP1 SPINK4 FBXO17 PLEKHM1 TAPBP CREB3L4 SSR1 DAXX SEC61A2 RAPSN RPS17 RPS17L NMT1

c

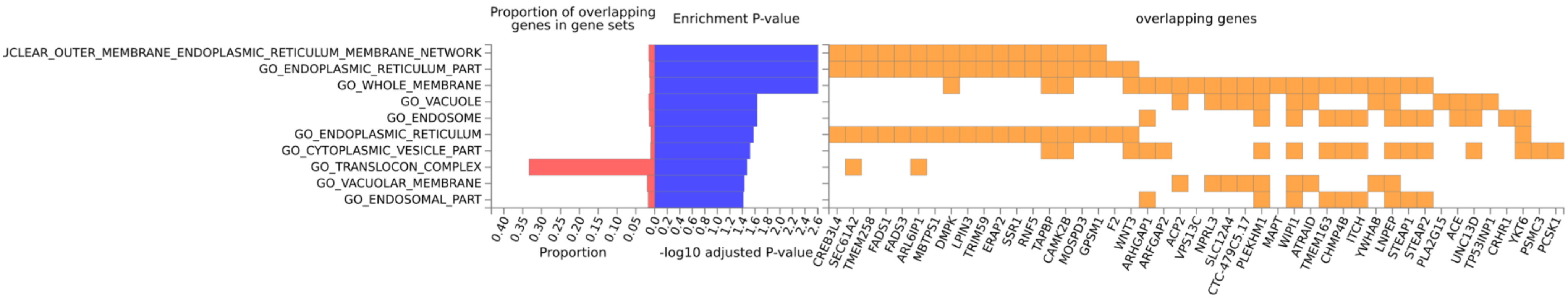

Supplementary Figure 6. Overrepresentation of biological functions of SMR prioritized genes for metaGLU (number of genes=185) tested against GO gene sets obtained from MsigDB (**a**) GO molecular functions (**b**) GO biological processes and (**c**) GO cellular components using hypergeometric tests. FUMA reports all gene sets with FDR corrected *P*-value < 0.05 (Benjamini–Hochberg method).

a

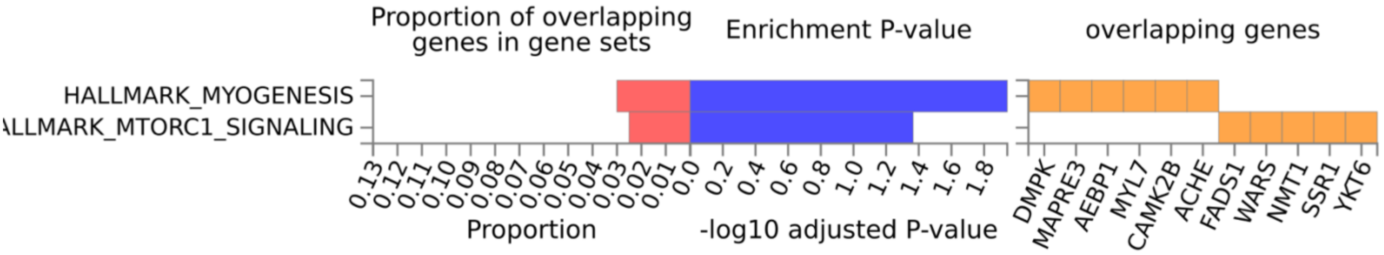

b

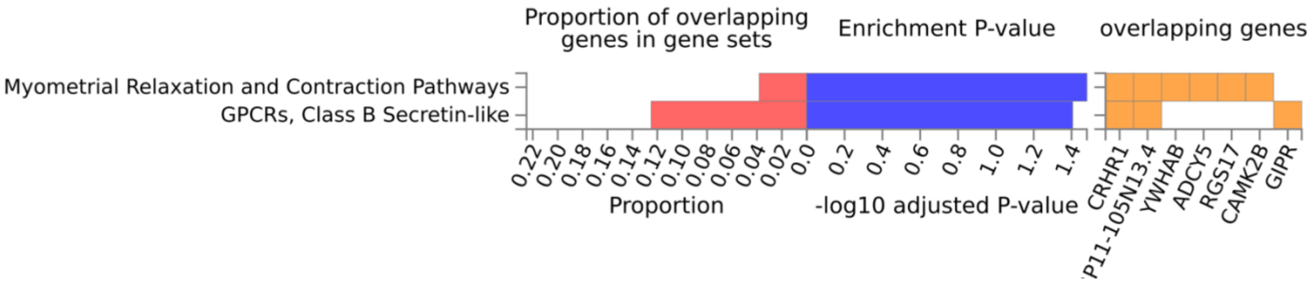

Supplementary Figure 7. Overrepresentation of biological functions of SMR prioritized genes for metaGLU (number of genes=185) tested against gene sets obtained from (**a**) MsigDB (hallmark gene sets) and (**b**) WikiPathways using hypergeometric tests. FUMA reports all gene sets with FDR corrected *P*-value < 0.05 (Benjamini–Hochberg method).

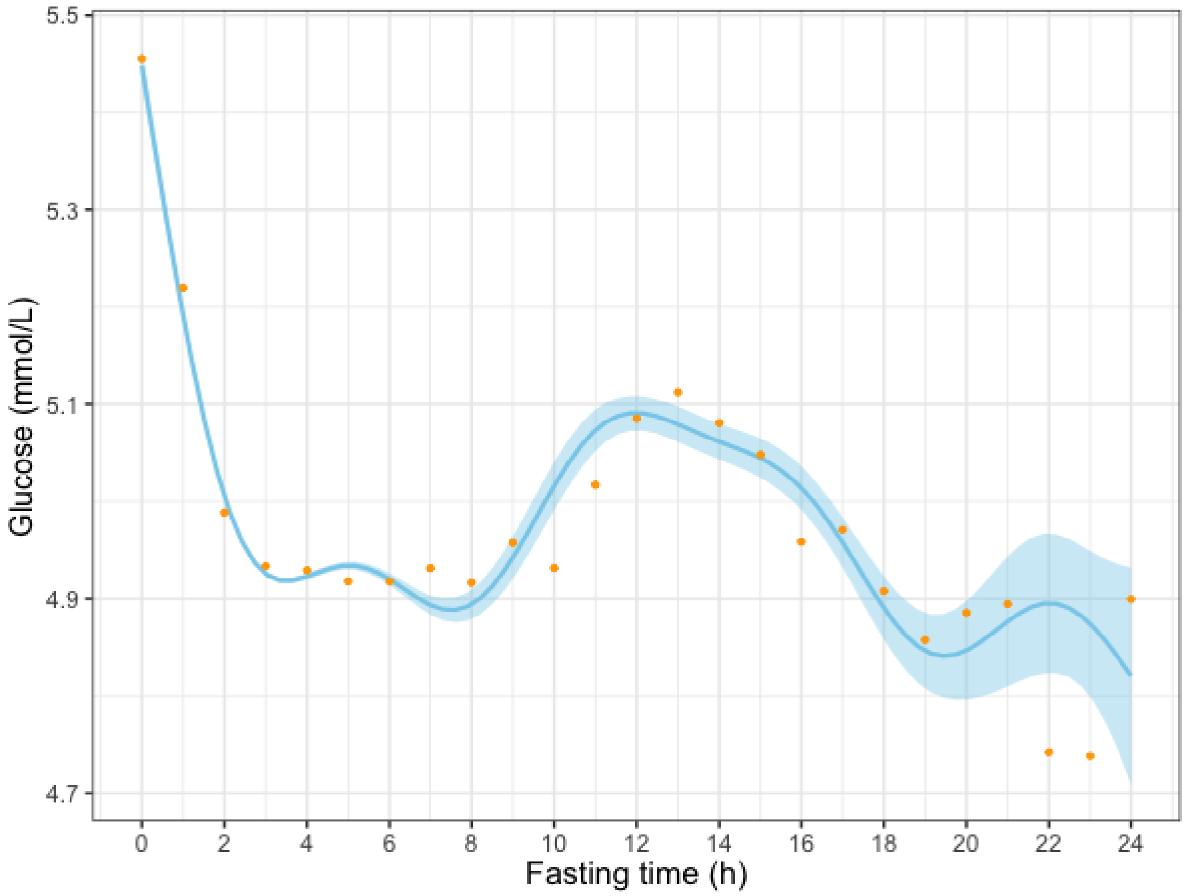

Supplementary Figure 8. The shape of post-prandial glucose curves among UKB participants (*N*=367,427). The centre line represents the general additive model spline and the shaded region is the 95% confidence interval. The orange point represents the mean glucose levels in each fasting time interval.

ACKNOWLEDGEMENTS

LifeLines Cohort Study

The LifeLines Cohort Study, and generation and management of GWAS genotype data for the LifeLines Cohort Study is supported by the UMCG Genetics Lifelines Initiative (UGLI), the Netherlands Organization of Scientific Research NWO (grant 175.010.2007.006), the Economic Structure Enhancing Fund (FES) of the Dutch government, the Ministry of Economic Affairs, the Ministry of Education, Culture and Science, the Ministry for Health, Welfare and Sports, the Northern Netherlands Collaboration of Provinces (SNN), the Province of Groningen, University Medical Center Groningen, the University of Groningen, Dutch Kidney Foundation and Dutch Diabetes Research Foundation.
The authors wish to acknowledge the services of the Lifelines Cohort Study, the contributing research centers delivering data to Lifelines, and all the study participants.

GROUP AUTHORS

LifeLines group author genetics

LifeLines Cohort Study
Raul Aguirre-Gamboa[1], Patrick Deelen[1], Lude Franke[1], Jan A Kuivenhoven[2], Esteban A Lopera Maya[1], Ilja M Nolte[3], Serena Sanna[1], Harold Snieder[3], Morris A Swertz[1], Judith M Vonk[3], Cisca Wijmenga[1]
[1]Department of Genetics, University of Groningen, University Medical Center Groningen, The Netherlands
[2]Department of Pediatrics, University of Groningen, University Medical Center Groningen, The Netherlands
[3]Department of Epidemiology, University of Groningen, University Medical Center Groningen, The Netherlands

SUPPLEMENTARY REFERENCES

1. Yengo, L. *et al.* Meta-analysis of genome-wide association studies for height and body mass index in approximately 700000 individuals of European ancestry. *Hum Mol Genet* **27**, 3641-3649 (2018).
2. Lagou, V. *et al.* Sex-dimorphic genetic effects and novel loci for fasting glucose and insulin variability. *Nat Commun* **12**, 24 (2021).
3. Yang, J. *et al.* Conditional and joint multiple-SNP analysis of GWAS summary statistics identifies additional variants influencing complex traits. *Nat Genet* **44**, 369-75, S1-3 (2012).
4. Vosa, U. *et al.* Large-scale cis- and trans-eQTL analyses identify thousands of genetic loci and polygenic scores that regulate blood gene expression. *Nature Genetics* **53**, 1300-+ (2021).
5. Bernstein, B.E. *et al.* The NIH Roadmap Epigenomics Mapping Consortium. *Nat Biotechnol* **28**, 1045-8 (2010).
