# Supplementary material for: Estimation and implications of the genetic architecture of fasting and non-fasting blood glucose
Source: Nat Commun. 2023 Jan 27;14:451. doi: 10.1038/s41467-023-36013-1 (PMC9883484; doi:10.1038/s41467-023-36013-1)
Supplement: Supplementary file 3 — Description of Additional Supplementary Files [file 41467_2023_36013_MOESM3_ESM.pdf]

Description of Additional Supplementary Files

File Name: Supplementary Data 1
Description: **ICD (WHO International Classification of Diseases) coding for diabetic diseases, and coding of self-reported diabetic-related medication or treatment use (Data-coding 4, Data-field 20003) in UKB.** ICD-9 coding is displayed in Data-Coding 87 (https://biobank.ndph.ox.ac.uk/showcase/coding.cgi?id=19&nl=1) and ICD-10 coding is displayed in Data-Coding 19 (https://biobank.ndph.ox.ac.uk/showcase/coding.cgi?id=87&nl=1) in UKB.

File Name: Supplementary Data 2
Description: **The descriptive statistics of all and unrelated individuals with non-missing genotype and phenotype data in each subgroup.**

File Name: Supplementary Data 3
Description: **SNP-based heritability estimates of random glucose (RG) estimated with different methods in each subgroup.**

File Name: Supplementary Data 4
Description: **Genetic correlation between fasting glucose (FG) and glucose levels in each subgroup estimated by the LDSC based on unrelated samples only. Genetic correlations were estimated using LDSC regression. Pairs of subgroups with genetic correlations significantly different from 1 (two-sided P-value < 0.05) indicate genetic heterogeneities between these subgroups.**

File Name: Supplementary Data 5
Description: **Results from univariate and bivariate LDSC anlysis (with standard errors) between pairwise subgroups based on all samples.**

File Name: Supplementary Data 6
Description: **Independent associations identified with GCTA-COJO analysis applied to GWAS summary statistics of fasting glucose (FG, pink fill), mega-GWAS of random glucose (mega-RG, yellow fill), meta-GWAS of random glucose (meta-RG, green fill), and meta-analysis of glucose (metaGLU, blue fill), respectively.**

File Name: Supplementary Data 7
Description: **The summary statistics of SNP *rs1881415* detected to have heterogeneity in genetic effects across fasting time in the current study.**

File Name: Supplementary Data 8
Description: **Genetic correlation between fasting glucose (FG), random glucose (RG) and other traits (*N*=245).** Genetic correlation (*rg*) was calculated between FG/RG and 245 traits (we manually replaced the GWAS summary statistics of T2D with a more recent study than that included in LD Hub). Significant genetic correlations were defined at the Bonferroni-corrected P-value < 2.04 × 10-4 (0.05/245). Note that several traits (trait 2) have a significant bivariate

LDSC intercept with FG/RG as these traits were estimated using UKB samples and therfore there exist sample overlaps.

File Name: Supplementary Data 9
Description: **Summary of SNP heterogeneity detected when meta-analysing FG with meta-RG.** SNPs are selected based on the following criteria: (1) reach genome-wide significance ($P <$ 5E-8) in either FG or meta-RG; (2) with *p*-value for heterogeneity (*PHET* < 5E-8); (3) is identified as **LD-independent through clumping (LD r2 < 0.01) in either FG or meta-RG GWAS.**

File Name: Supplementary Data 10
Description: **Supplement to Table 2.**

File Name: Supplementary Data 11
Description: **Significant associations in different tissues identified from SMR analyses on (a) fasting glucose (FG, Lagou et al. 2021); (b) mega-GWAS of random glucose (mega-RG); (c) meta-GWAS of random glucose (meta-RG); (d) meta-analysis of glucose (metaGLU), based on eQTL summary data from the eQTLGen, InsPIRE and GTEx consortium.**

File Name: Supplementary Data 12
Description: **Heritability and genetic correlations between fastinig time points for 30 blood and urine biomarkers available in UKB.**
