# Supplementary material for: Estimation and implications of the genetic architecture of fasting and non-fasting blood glucose
Source: Nat Commun. 2023 Jan 27;14:451. doi: 10.1038/s41467-023-36013-1 (PMC9883484; doi:10.1038/s41467-023-36013-1)
Supplement: Supplementary file 5 — Reporting Summary [file 41467_2023_36013_MOESM5_ESM.pdf]

nature portfolio

Corresponding author(s): Dr Loic Yengo

Last updated by author(s): Dec 17, 2022

Reporting Summary

Nature Portfolio wishes to improve the reproducibility of the work that we publish. This form provides structure for consistency and transparency in reporting. For further information on Nature Portfolio policies, see our Editorial Policies and the Editorial Policy Checklist.

Statistics

For all statistical analyses, confirm that the following items are present in the figure legend, table legend, main text, or Methods section.

n/a	Confirmed	
☐	☒	The exact sample size (*n*) for each experimental group/condition, given as a discrete number and unit of measurement
☒	☐	A statement on whether measurements were taken from distinct samples or whether the same sample was measured repeatedly
☐	☒	The statistical test(s) used AND whether they are one- or two-sided<br>*Only common tests should be described solely by name; describe more complex techniques in the Methods section.*
☐	☒	A description of all covariates tested
☐	☒	A description of any assumptions or corrections, such as tests of normality and adjustment for multiple comparisons
☐	☒	A full description of the statistical parameters including central tendency (e.g. means) or other basic estimates (e.g. regression coefficient) AND variation (e.g. standard deviation) or associated estimates of uncertainty (e.g. confidence intervals)
☐	☒	For null hypothesis testing, the test statistic (e.g. *F*, *t*, *r*) with confidence intervals, effect sizes, degrees of freedom and *P* value noted<br>*Give P values as exact values whenever suitable.*
☒	☐	For Bayesian analysis, information on the choice of priors and Markov chain Monte Carlo settings
☒	☐	For hierarchical and complex designs, identification of the appropriate level for tests and full reporting of outcomes
☐	☒	Estimates of effect sizes (e.g. Cohen's *d*, Pearson's *r*), indicating how they were calculated

Our web collection on statistics for biologists contains articles on many of the points above.

Software and code

Policy information about availability of computer code

Data collection	No software were used for data collection.
Data analysis	Statistical analyses were performed mostly with R version v4.21, PLINK v1.90b6.11 and PLINK v2.00aLM, GCTA v1.93.1.beta, BOLT-LMM vs 2.3.4, METAL (version 2011-3-25)

For manuscripts utilizing custom algorithms or software that are central to the research but not yet described in published literature, software must be made available to editors and reviewers. We strongly encourage code deposition in a community repository (e.g. GitHub). See the Nature Portfolio guidelines for submitting code & software for further information.

Data

Policy information about availability of data

All manuscripts must include a data availability statement. This statement should provide the following information, where applicable:

- Accession codes, unique identifiers, or web links for publicly available datasets
- A description of any restrictions on data availability
- For clinical datasets or third party data, please ensure that the statement adheres to our policy

We used genotypic data and phenotypic data from the UK biobank under project number 12505. UKB data can be accessed upon request once a research project has been submitted and approved by the UKB research committee. We also used genotypes and phenotypes from the Lifelines Study. Each dataset is described in the method section.

All datasets used in this study are available in the public domain. This study uses genotype and phenotype data from UK Biobank Resource under project 12505. UKB data can be accessed upon request once a research project has been submitted and approved by the UKB committee. Data on glycaemic traits were downloaded from www.magicinvestigators.org. Other datasets used in these analyses can be sourced from: eQTLGen Consortium Data, http://www.eqtlgen.org/cis-eqtls.html. GERA, https://www.ncbi.nlm.nih.gov/projects/gap/cgi-bin/study.cgi?study_id=phs000674.v2.p2. GTEx, https://gtexportal.org/home/datasets. HapMap3, https://www.sanger.ac.uk/resources/downloads/human/hapmap3.html. Lifelines cohort study, https://www.lifelines.nl/researcher. Banded LD matrix of ~1.1 million HapMap3 SNPs computed from 10,000 unrelated UKB individuals of European ancestry: https://cnsgenomics.com/software/gctb/#Download.

Genome-wide association summary statistics generated from this study (i.e., mega-GWAS of random glucose, meta-GWAS of random glucose, meta-analysis of glucose) are available for download from https://cnsgenomics.com/data/qiao_et_al_2023_nc/.

Human research participants

Policy information about studies involving human research participants and Sex and Gender in Research.

Reporting on sex and gender	We used sex a covariate to adjust our analyses. There is no mention of gender in this study.
Population characteristics	We describe the distribution of glucose (mean and variance) in Supplementary Table 2. We performed a GWAS of random glucose corrected for age at recruitment, sex, genotyping batches, assessment centre, and 40 genotypic principal components to account population stratification.
Recruitment	UK Biobank investigators sent postal invitations to 9,238,453 individuals registered with the UK's National Health Service who were aged 40–69 years and lived within approximately 25 miles (40 km) of one of 22 assessment centers located throughout England, Wales, and Scotland. Overall, 503,317 participants consented to join the study cohort and visited an assessment center between 2006 and 2010, resulting in a participation rate of 5.45%. (Fry et al. Am J Epidemiol. 2017 Nov 1;186(9):1026-1034).
Ethics oversight	The National Information Governance Board for Health and Social Care and the North West Multicentre Research Ethics Committee provided approval for UK Biobank to obtain the contact details of people within the eligible age range from local National Health Service Primary Care Trusts.

Note that full information on the approval of the study protocol must also be provided in the manuscript.

Field-specific reporting

Please select the one below that is the best fit for your research. If you are not sure, read the appropriate sections before making your selection.

☒ Life sciences ☐ Behavioural & social sciences ☐ Ecological, evolutionary & environmental sciences

For a reference copy of the document with all sections, see nature.com/documents/nr-reporting-summary-flat.pdf

Life sciences study design

All studies must disclose on these points even when the disclosure is negative.

Sample size	We used pre-existing data from the UK Biobank (N=487,371) and subset of the Lifelines study (N=13,781) with both phenotypes and genotypes. The sample size of unrelated European ancestry participants in the UK Biobank with available glucose measures used in this study (N=280,962) allows us to detect, with >90% of statistical power, associations with variants explaining at least 0.017% of glucose variance. The sample of the Lifelines samples used in this study (N=13,781) allows to detect (with >90% statistical power) correlations between polygenic scores and glucose as large as 6%, and also to estimate heritability with an expected standard error of 2.3%.
Data exclusions	We analysed European descent participants in the UK Biobank (Sample size left after exclusion: N=367,427) and Lifelines (Sample size left after exclusion: N=13,781)without diabetes. We excluded individuals under 18 (in particular in Lifelines) and those with a fasting glucose >=7mmol/L or an random glucose value >=11 mmol/L.
Replication	Replication (prediction analyses) were performed in a sample of 13,781 European participants from the Lifelines study.
Randomization	N/A - Rationale: No intervention was implemented on study participants. We used all available data from European ancestry participants.
Blinding	N/A - Rationale: No intervention was implemented on study participants.

Reporting for specific materials, systems and methods

We require information from authors about some types of materials, experimental systems and methods used in many studies. Here, indicate whether each material, system or method listed is relevant to your study. If you are not sure if a list item applies to your research, read the appropriate section before selecting a response.

Materials & experimental systems

n/a	Involved in the study
☒	☐ Antibodies
☒	☐ Eukaryotic cell lines
☒	☐ Palaeontology and archaeology
☒	☐ Animals and other organisms
☒	☐ Clinical data
☒	☐ Dual use research of concern

Methods

n/a	Involved in the study
☒	☐ ChIP-seq
☒	☐ Flow cytometry
☒	☐ MRI-based neuroimaging
